# Mediterranean Plants and Spices as a Source of Bioactive Essential Oils for Food Applications: Chemical Characterisation and *In Vitro* Activity

**DOI:** 10.3390/ijms26083875

**Published:** 2025-04-19

**Authors:** Federica Barbieri, Giulia Tabanelli, Giacomo Braschi, Daniela Bassi, Sara Morandi, Vida Šimat, Martina Čagalj, Fausto Gardini, Chiara Montanari

**Affiliations:** 1 Department of Agricultural and Food Sciences, University of Bologna, 47521 Cesena, Italy; federica.barbieri16@unibo.it (F.B.); giacomo.braschi2@unibo.it (G.B.); fausto.gardini@unibo.it (F.G.); chiara.montanari8@unibo.it (C.M.); 2 Department of Agricultural and Food Sciences, University of Bologna, 40127 Bologna, Italy; 3 Department for Sustainable Food Process (DISTAS), Università Cattolica del Sacro Cuore, 26100 Cremona, Italy; daniela.bassi@unicatt.it (D.B.); sara.morandi2@unicatt.it (S.M.); 4 Department of Marine Studies, University of Split, HR-21000 Split, Croatia; vida@unist.hr (V.Š.); mcagalj@unist.hr (M.Č.)

**Keywords:** essential oils, antimicrobial activity, antioxidant activity, bioactive compounds, officinal plants, spices

## Abstract

Mediterranean officinal plants and spices are rich sources of bioactive compounds that can be used to improve the safety and quality of fresh food products. Among them, essential oils (EOs), known for their antimicrobial and antioxidant activities, can be a feasible solution for natural and healthy foods with low environmental impact. This study evaluates the bioactive potential of ten EOs derived from Mediterranean officinal plants and spices. Gas Chromatography-Mass Spectrometry (GC-MS) analysis identified compounds with known antioxidant and antimicrobial properties. *In vitro* antioxidant potential using different methods highlighted the promising effect of cloves and cinnamon EOs. Minimum Inhibitory Concentration (MIC) assays revealed strong antimicrobial activity of oregano and cinnamon EOs against foodborne pathogens, including *Listeria monocytogenes* and *Staphylococcus aureus*, with MIC values ranging from 0.25 to 0.50 mg/mL. This activity can be attributed to the predominance of carvacrol and cinnamaldehyde, whose antimicrobial activity is well-documented. The impact of medium pH and water activity on the antimicrobial efficacy of the EOs was also assessed. Overall, this research contributes to increasing the knowledge of the potential applications of plant-derived EOs in food preservation, offering a natural, sustainable, and consumer-friendly approach to enhancing food quality, safety, and nutritional value.

## 1. Introduction

The increasing negative consumer perceptions of synthetic food additives have directed research on using natural and eco-friendly preservatives as a green and sustainable alternative solution in the food industry. In this perspective, particular attention has focused on the application of essential oils (EOs), obtained from officinal plants and spices to enhance the microbial safety and chemical stability of products [1,2]. Many of these compounds have been generally recognized as safe (GRAS) by the Food and Drug Administration (FDA) for consumer use [3]. Essential oils are a complex combination of numerous volatile molecules, mainly terpenes, terpenoids, and phenylpropenes, present at different concentrations [4,5]. These substances, responsible for the intense flavour and aroma, can be characterised by strong antioxidant and antimicrobial activities against spoilage microorganisms or food-borne pathogens [6]. EOs can exert bacteriostatic or bactericidal effects, depending on their chemical composition. However, their mechanisms of action are not well known [7]. Nevertheless, EOs antimicrobial properties can be influenced by synergic and complex interactions between different compounds and often are not dependent on a simple additive effect of the single molecules present [8,9]. In general, the antimicrobial action of EOs molecules is firstly based on their ability to solubilize into the microbial phospholipidic cell membranes due to their hydrophobicity, being Gram-positive bacteria more sensitive to EOs effect with respect to Gram-negative ones, more protected by the outer membrane [1]. As an immediate consequence, the fluidity of the membrane may be altered, with possible impairment of molecules associated with the membranes themselves. Finally, membrane permeability can be affected, compromising some relevant functional properties of the cell, i.e., membrane potential, enzymatic activities, and transport systems [5,10].

EOs chemical composition of different plant species and the consequent antimicrobial effect and antioxidant effect, are highly affected by many different factors such as the chemotype/cultivar, the part of the plant (flowers, roots, seeds, leaves, bark, etc.) used to obtain the EOs extract, the geographical area, the agronomic and meteorological conditions, the harvesting season, and the extraction method (hydro-distillation, steam distillation, cold pressing, supercritical CO_2_, etc.) [6,11]. In addition, other factors influence EO antimicrobial properties, like specific food matrices or food components (fats, proteins, water, salt, etc.) that can reduce the EO availability and effects [5,12]. In addition, intrinsic factors (pH, aw, temperature, etc.) can modify EO bioactive properties due to the effect exerted on the functional chemical groups. Although limited literature is available on this topic, some studies have indicated that the sensitivity of microorganisms to certain EOs is enhanced by a reduction in pH and water activity (a_w_), which affects the solubilization of EOs into cell membranes [7,12,13,14].

The bioactive compounds profile of Mediterranean plants and spices EOs constitutes of polyphenols, flavonoids, and terpenoids, all found responsible for their antioxidant potential. Besides being used to enhance food flavour and aroma or for aromatherapy, the volatile compounds from EOs are known to inhibit free radicals and minimise oxidative stress, a feature that makes them interesting for a wide range of food applications as ingredients and flavouring agents. In particular, clove essential oil exhibits strong antioxidant activity, primarily due to its high eugenol content [15,16], while oregano EO shows antioxidant potential due to carvacrol and thymol [17]. The antioxidant activity of laurel and sage EOs was reported due to their dominant compounds, eucalyptol and rosmarinic acid [16,18]. Fennel EO, rich in trans-anethole, has also been known for its antioxidant properties [19,20]. These findings show the importance of essential oils as natural alternatives to synthetic antioxidants in various industries.

Based on the outlined background, this study aims to characterise and evaluate the *in vitro* antimicrobial and antioxidant activities of ten commercial EOs derived from Mediterranean plants and spices, including cinnamon, cloves, cumin, fennel, juniper, laurel, marjoram, myrtle, oregano, and sage. EO chemical characterization was assessed by Gas Chromatography-Mass Spectrometry (GC-MS) analysis, while the antimicrobial activity against *Listeria monocytogenes* Scott A, *Staphylococcus aureus* DSM 20231^t^, *Escherichia coli* 555 and *Enterococcus faecalis* EF37 was studied through the determination of minimum inhibiting concentration (MIC) and minimum bactericidal concentration (MBC). To explore the influence of chemico-physical parameters on EO bioactivity, these analyses were conducted at varying pH levels (7, 6, and 5) and salt concentrations (0%, 3%, and 5%). The antioxidant potential of the EOs was measured using three different assays. Two based on the hydrogen atom transfer (2,2-diphenyl-1-picrylhydrazyl (DPPH) radical scavenging activity; oxygen radical absorbance capacity (ORAC)) and a ferric reducing antioxidant power (FRAP) method that is based on the electron transfer mechanism.

## 2. Results and Discussion

### 2.1. Characterization of EOs Deriving from Mediterranean Plants and Spices

The chemical composition of the EOs extracted from 10 different Mediterranean plants is summarized in Table 1. About 80 molecules were identified, mainly represented by terpenes, terpenoids and phenylpropenes. To facilitate comprehension, only the compounds that were present at 0.5% in at least one of the samples are reported.

As expected, the EO profiles varied depending on the plant origin, with some of them characterized by a few molecules detected in high amounts.

Concerning cinnamon (*Cinnamomum zeylanicum*) EO, about 75% of the total peak area was represented by cinnamaldehyde (62%), followed by cinnamyl acetate (14%) and eugenol (4.5%). This reflects data reported by other Authors, in which cinnamaldehyde was the predominant compound [21,22], even if the latter also observed a notable amount of alpha-pinene (about 10%), probably deriving from cinnamon bark, that in the present study was almost negligible (1%). Eugenol was the molecule characterizing clove EO (75% of the total peak area), followed by its acetate ester (18.5%) and caryophyllene (4.5%), while other few molecules were detected in very low amounts (less than 1%). Other papers indicated that clove (*Syzygium aromaticum*) EO is generally constituted by about 30 compounds, depending also on the extraction method, among which eugenol accounts for at least half of the total composition [23,24].

Remaining in the family of *Myrtaceae*, a more complex composition has been detected in myrtle (*Myrtus communis*) EO, characterized by the predominance of eucalyptol (34%), followed by α-pinene, myrtenyl acetate, D-limonene, linalool, α-terpineol and geranyl acetate. The same compounds were reported in a study on the composition and antimicrobial and antioxidant activities of *Myrtus communis* EOs derived from different countries and obtained using different parts of the plants [25]. Considering EOs extracted from flowers, i.e., the same matrix of the present study, the qualitative composition was similar, even if the relative percentages were different in relation to the geographical origin, with eucalyptol generally not exceeding 25%, and reaching higher contents (up to 50%) only when leaves were used for EO extraction [26]. Eucalyptol was also the compound detected in a higher percentage (about 39% of the total peak area) in the laurel (*Laurus nobilis*) EO, which was also characterized by a significant presence of terpineol acetate (almost absent in all the other tested samples), β-phellandrene, linalool, α-pinene, eugenol, and β-pinene. A similar composition was found by other authors who compared the chemical profiles of laurel leaf derivatives (phenolic extracts and EOs) collected in Greece and Georgia [27]. In both EOs, the main constituent was eucalyptol (about 30%), while the ratio of other compounds was different in relation to the geographical origin. Indeed, in the present study, the terpineol acetate amount was about 15%, thus in agreement with values reported for Greek laurel plants, but lower with respect to Georgian ones (about 23%) [27]. Beta-phellandrene, which accounted for about 10% of the total peak area in our EO, was not detected in the study previously cited. Conversely, α-pinene and β-pinene presented similar values (ranging from 4 to 5% and from 3 to 4%, respectively) independently of the tested EO. As expected, in cumin (*Cuminum cyminum*) EO the main compound detected by GC-MS analysis was cuminaldehyde (about one-third of the total peak area), while the remaining part was represented by ɤ-terpinen-7-al (15%), β-pinene (13%), ɤ-terpinene (13%), p-cymene (12)% and α-terpinen-7-al (10%), already reported in literature as primary bioactive compounds of such EO [28,29].

Another seed-derived EO analyzed in the present study was fennel (*Foeniculum vulgare*) EO, in which anethole accounted for half of the total peak area, followed by α-pinene (13%), α-phellandrene (12%), limonene (10%) and fenchone (5%). The composition of fennel EO can significantly vary in relation to the part of the plant used (stem, leaves, umbels) and the maturation stages. In general, anethole is always the predominant compound (with concentrations ranging from 50 to 88% mainly depending on raw material), while minor compounds such as those reported above are more variable also in relation to the plant maturation stage [30,31].

Among Mediterranean plants that can be exploited as a source of bioactive compounds, juniper is surely worth mentioning. The genus *Juniperus* includes many species, among which *Juniperus communis* is one of the most widespread. Also, in this case, the chemical profiles vary with geographical and seasonal factors, as well as the part used for extraction (needles vs. fruits) [4], but usually, the main constituent is α-pinene, which in this study accounted for about 31% of the total peak area. Other compounds detected were β-phellandrene (15%), β-myrcene (15%), limonene (3.5%), β-copaene (7%), and δ-cadinene (4%). Some of them were also found in EOs obtained in Portugal from *J. communis* berries, but with different concentrations depending on the origin (commercial samples or laboratory-hydro distilled EOs) [32].

The last family considered in this screening was *Lamiaceae*, typical of the Mediterranean basin and including more than 5000 species, some of which are used as ornamental and edible plants, but also as a source to obtain EOs characterized by strong antioxidant and antibacterial properties [33]. In particular, three commercial EOs obtained from flowering tops of *Origanum vulgare*, *Origanum majorana* and *Salvia officinalis* were analyzed. The results in Table 1 show that marjoram was mainly characterized by terpinen-4-ol (24%), β-terpineol, ɤ-terpinene and β-phellandrene. Other studies reported a significant amount of cis and trans-sabinene hydrate [34], which was not detected here: this can be explained by the presence of different chemotypes. Indeed, research focused on individual plants of sweet marjoram grown in Cyprus identified three distinct chemotypes: a “marjoram typical” sabinyl-chemotype (characterized by the predominance of sabinyl compounds), a “pure” α−terpineol chemotype (73% of α−terpineol) and a mixed chemotype [35].

The composition of origanum EO was mainly represented by carvacrol (about 76%), followed by p-cymene (7%, precursor of thymol), ɤ-terpinene (5%, precursor of carvacrol), and thymol (less than 3%). A significant predominance of carvacrol with respect to other molecules was also observed in some EOs collected in South Italy [36]. Those Authors analyzed EOs deriving from 25 wild populations of *Origanum vulgare* grown in different locations of the Calabria Region, highlighting the presence of different chemotypes characterized by differences in the amounts of specific compounds such as thymol, carvacrol, and linalyl acetate. For example, high thymol content was associated with higher altitude, suggesting the activation of biosynthetic pathways to adapt to environmental variations. Also, oregano EO from Portugal showed the occurrence of thymol, carvacrol, and ɤ-terpinene as major constituents [37]. Finally, concerning sage EO, one-third of the total peak area was represented by thujone, followed by camphor (12%), eucalyptol (11%), humulene (8%), and camphene, caryophyllene, α-pinene, and β-pinene (about 4% each).

The same compounds have been identified in *Salvia officinalis* EOs originating from different countries (e.g., Montenegro, China, Spain, and Albania), although their relative abundances varied depending on the geographical origin and the specific plant part used for extraction (leaves or flowers) [38].

Thujone in general ranged from 15 to 50%, with higher values when flowers were used for the extraction. Indeed, it was not detected in an EO obtained from a sage herb in Turkey [39], confirming that its presence can derive from the flowering part of the plant. The percentage of camphor was more variable (in some cases also absent), with the highest values detected in the samples collected in south Albania [40].

### 2.2. In Vitro Antioxidant Potential

**Figure 1:**
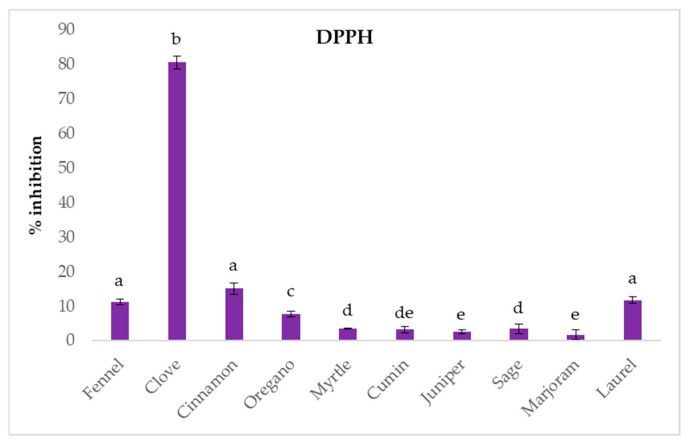
The results of DPPH inhibition (%) for tested essential oils. Columns marked with different letters are statistically different (*p* < 0.05).

The *in vitro* antioxidant potential was determined using DPPH radical scavenging ability, FRAP and ORAC method. The results for the DPPH radical inhibition (Figure 1) of the analyzed EOs showed that clove EO exhibited the highest antioxidant activity with 80.45 ± 1.86% inhibition, significantly higher than other oils. Cinnamon EO showed moderate inhibition at 15.14 ± 1.60%, followed by fennel (11.30 ± 0.85%) and laurel (11.82 ± 0.86%). Oregano EO exhibited a lower inhibition of 7.77 ± 0.90%, while the remaining samples, including myrtle, cumin, juniper, sage, and marjoram, showed low DPPH radical inhibition of less than 4%.

These results show that clove EO had the most potent antioxidant activity among the tested samples. Similar results were reported by other Authors [15], with clove EO exhibiting high DPPH radical inhibition, nearly 100% of inhibition at 200 µg/mL, surpassing even synthetic antioxidants like BHT at similar concentrations. However, for oregano EO, the same authors found a DPPH inhibition of 20.25% at the same concentration. For fennel oil, Shahat et al. [19] investigated the antioxidant activity of various fennel cultivars and reported IC50 values ranging from 0.35 to 15.33 mg/mL, which showed variation in antioxidant potential across tested types. Similar to our results, exceptional DPPH activity, with an EC50 value of 0.008 µg/mL, was found for clove EO by other Authors [41]. Furthermore, laurel EO showed moderate DPPH inhibition with an EC50 of 0.680 µg/mL, consistent with our results. Other EOs like cumin, sage, and juniper, did not show significant DPPH scavenging activity.

**Figure 2:**
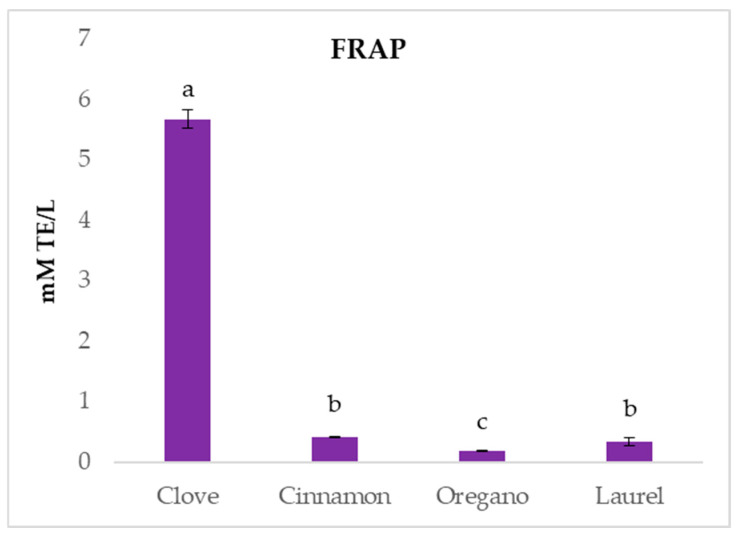
The FRAP results for tested essential oils. Columns marked with different letters are statistically different (*p* < 0.05).

The FRAP assay results (Figure 2) showed that clove EO had the highest reducing activity, with a value of 5.67 ± 0.15 mM TE/L, significantly higher than all other tested samples. Other EOs such as cinnamon, oregano and laurel had low reducing activity. Similar results were reported by other Authors [15], which evidenced for clove oil significantly higher FRAP values than oregano and sage oils, attributing its activity to the high eugenol content. Furthermore, Mladenović et al. [41] also reported that clove EO possessed excellent ferric-reducing potential while oregano, cinnamon and laurel EO had moderate activity.

**Figure 3:**
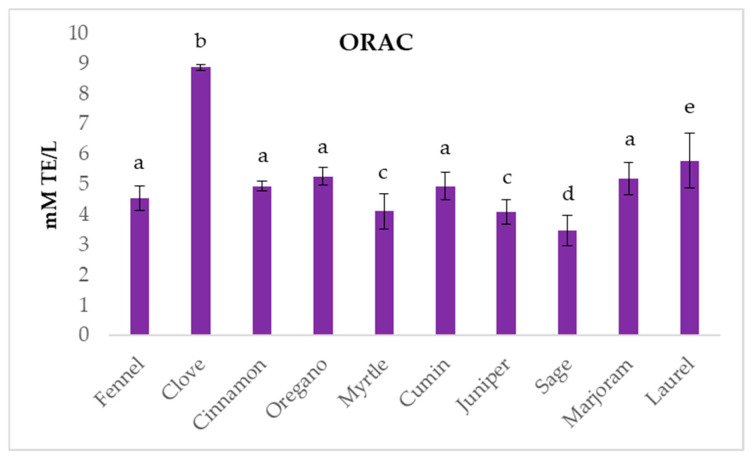
The ORAC results for tested essential oils. Columns marked with different letters are statistically different (*p* < 0.05).

The ORAC assay results (Figure 3) showed that clove EO had the highest antioxidant activity with 8.88 ± 0.10 mM TE/L. Laurel EO had the second-best result with a value of 5.79 ± 0.90 mM TE/L, while oregano, marjoram, and cinnamon showed similar activity that ranged from 5.26 ± 0.29 to 3.48 ± 0.51 mM TE/L. The lowest result was recorded for sage EO. Overall, the comparison of the results obtained from the three antioxidant assays used evidenced that clove EO was the strongest antioxidant among the tested EOs.

### 2.3. Antimicrobial Activity of EOs Against Food-Borne Pathogens/Toxigenic Bacteria

To evaluate the antimicrobial activity of the different EOs, MIC and MBC against *L. monocytogenes*, *Staph. aureus*, *E. coli* and *Ent. faecalis* were assessed. The results regarding MIC and MBC in relation to pH are summarized in Table 2.

In certain cases, the MIC could not be determined, as the highest tested concentration (5 mg/mL) did not exhibit inhibitory effects on the target microorganisms. This was observed for fennel, juniper, and myrtle essential oils, where no inhibition was recorded, regardless of the medium pH or the type of microorganism. These data partially disagree with those previously reported for fennel EO, where some authors highlighted a MIC value of 250 m/L against *E. coli*, even if this EO was not active against *Staph. aureus* (MIC > 10,000 mg/L) [42].

Concerning myrtle, its antioxidant and antibacterial activity have been recently reviewed [25], and a wide array of MIC values were reported for bacteria (from few to thousands of ppm), but in general the most promising results were obtained with EOs extracted from leaves, while in the present study flowering tops were used. Also, for juniper, a previous study with *Juniperus communis* berries EO evidenced MIC values strongly variable in relation to the type of EO (commercial or laboratory hydrodistilled) and the pathogen tested [32]. For example, for *Staph. aures* MIC ranged from 1.6 mg/mL to 6.3 mg/mL, while for *E. coli* and *Ent. faecalis* inhibiting concentrations were generally 3.1 mg/mL.

Sage EO was active only against *Staph. aureus*, with a MIC value of 750 mg/L, corresponding also to MBC, that slightly increased by decreasing medium pH. This sensitivity of *Staph. aureus* was not recorded in previous studies, in which the values reported for *Salvia officinalis* EOs ranged from 2.87 to 4.5 mg/mL [38] or were even higher than 25 mg/mL [39].

Laurel EO was active only in the more acidic condition tested (MH acidified at pH 5) against *Staph. aureus* (MIC and MBC values at 2 mg/mL) and, to a lesser extent, against *L. monocytogenes* Scott A (MIC value 4 mg/mL). Another study concerning different laurel derivatives (EOs and extracts) evidenced lower inhibiting concentrations for the latter pathogen (1.1 mg/mL for EO) and also activity against *E. coli*, with the same MIC value [43].

Also, marjoram EO showed higher antimicrobial activity against *Staph. aureus* at the lowest pH tested (MIC 1 mg/mL), while no effect was detected for *Ent. faecalis*. A slight activity was observed for *E. coli*, with MIC at 3 mg/mL, independently of the pH, and the same value was observed for *L monocytogenes*, but only at pH 5. A previous work focused only on different wild strains of *Staph. aureus* highlighted higher MIC values, ranging from 6.25 to 50 mg/mL [44].

Cumin EOs did not exert any activity on *Ent. faecalis* and *E. coli* and was able to inhibit *L. monocytogenes* only in acidified medium (MIC corresponding to 2 mg/mL at pH 5), even if it was not possible to find MBC in the tested condition (MBC > 5 mg/mL). However, a good antimicrobial effect was recorded again for *Staph. aureus*, with concentration decreasing from 4 to 1 mg/mL in relation to pH reduction. Other studies confirmed a higher sensitivity of this species if compared to other microorganisms such as *E. coli*, *Bacillus cereus*, and *Pseudomonas aeruginosa* [45,46].

Coming to the most promising EOs, cloves were active against all the target microorganisms: for *E. coli* MIC and MBC values were 1 mg/mL independently of pH, while for *Ent. faecalis* this concentration was reached only in acidified medium. *L. monocytogenes* and *Staph. aureus* were more sensitive, and MIC was strongly affected by pH: indeed, starting from 1 mg/mL in MH at pH 7, MIC value decreased down to 0.25 mg/mL at pH 5 for both pathogens. For the latter, this value also corresponded to MBC. Previous literature reported a wide range of concentrations exerting antagonist activity against spoilage or pathogen microorganisms, with differences depending on the type of derivative (EO, powder, water extracts, ethanol extracts) and the procedure adopted [28]. Some studies focused on clove EO reported MIC values against *Staph. aureus* of 0.625–0.780 mg/mL, while they were higher than 1.5 mg/mL for *Listeria innocua* and some Gram-negative bacteria [47,48].

Also, cinnamon was effective against all the tested microorganisms (MIC corresponding to 0.50 mg/mL for *Ent. faecalis* and 0.25 mg/mL for the other strains), without differences in relation to medium pH, except for *L. monocytogenes*, in which the lowest MIC value among all the tested EOs (0.10 mg/mL) was observed at pH 5. These findings are consistent with those of previous studies, showing MIC values ranging from 0.25 to 1 mg/mL for *E. coli*, while *Staph. aureus* was more susceptible, with MIC between 0.10 and 0.25 mg/mL [21]. Finally, the antimicrobial effect of oregano EO was slightly affected by pH, with values ranging from 0.40 mg/L to 0.25 mg/mL for *Ent. faecalis* and *E. coli*, while higher inhibitory activity was observed against *L. monocytogenes* (MIC 0.20 mg/mL) and, even more, against *Staph. aureus*, with MIC and MBC assessed at 0.15 mg/mL in acidified medium. The bioactivity of *Origanum vulgare* EO has been recently reviewed [49] and, comparing the results of the present study with those reported in that manuscript, data were quite similar for *E. coli* (MIC 0.16–0.60 mg/mL), lower for *L. monocytogenes* (MIC ranging from 0.32 to 1.20 mg/mL depending on the strain) and significantly different for *Ent. faecalis*, with MIC corresponding to 8 mg/mL, likely due to the low carvacrol content (about 12%) in the EO used for the trial. For *Staph. aureus*, a wide range of values was reported (0.08–1.19 mg/mL) in relation to the strain and the characteristics of the EO (chemical profile and origin).

The results regarding MIC and MBC in relation to NaCl concentration, i.e., different a_w_ values, are summarized in Table 3. In general, the presence of NaCl less affects EO antimicrobial activity if compared to medium acidification. For fennel, juniper and myrtle, no MIC was detected (values > 5 mg/mL, i.e., the maximum concentration tested), independently of a_w_. Laurel showed MIC and MBC at 0.25 mg/mL only against *E. coli* when grown with 5% NaCl.

A similar behaviour for this pathogen was observed for cumin, with MIC and MBC assessed at 2 mg/mL when NaCl was present, independently of its concentration. *E. coli* was the only tested microorganism sensitive also to marjoram EO: indeed, only in this case were MICs assessed in the tested concentration range, with values decreasing from 3 to 0.50 mg/mL by increasing salt concentration. Conversely, sage was active only against *Staph. aureus*, and MIC was almost halved at the highest NaCl concentration (5%). As reported for pH (Table 2), the most promising EOs were cloves, cinnamon and oregano. For cloves, a reduction of MIC concentration was observed at increasing NaCl amounts (from 1 to 0.20–0.30 mg/mL) for all the tested microorganisms, except for *Ent. faecalis*. Concerning cinnamon EO, an effect of water activity was found, in particular for *E. coli*, with a MIC of 0.10 mg/mL (i.e., more than halved with respect to the corresponding sample without NaCl). Oregano EO bioactivity against *Ent. faecalis* and *Staph. aureus* was not affected by water activity, while for the other strains, a significant effect was observed: in the case of *L. monocytogenes*, MIC passed from 0.25 to 0.15 mg/mL, while for *E. coli* this phenomenon was even more evident (MIC from 0.40 to 0.10 mg/mL).

The efficacy of some of the tested EOs can be attributed to their main constituents. In the cases of oregano, cinnamon, and clove oils, a single compound—carvacrol, cinnamaldehyde, and eugenol, respectively—accounted for at least two-thirds of the total peak area. The antimicrobial activity of these compounds has been extensively documented in both model systems and real-world applications [50].

The main component of oregano EOs is carvacrol, whose antibacterial effect has been recently reviewed [51,52]. Its mechanism of action primarily involves disruption of the bacterial cell membrane, resulting in cell lysis, leakage of intracellular contents, and ultimately, cell death. This effect is attributed to the hydrophobic nature of carvacrol, which facilitates its integration into the lipid bilayer of the membrane, thereby compromising membrane integrity, altering permeability, and leading to the dissipation of the proton motive force [53]. Other antibacterial mechanisms proposed in literature for this compound present in EOs include the reduction of biofilm formation, the inhibition of ATPase activity and efflux pumps, and the decrease of motility [51]. Carvacrol has been reported to be active against many food-borne pathogens such as *Staph. aureus*, *Salmonella* spp., *Shighella*, *Bacillus cereus*, *E. coli*, *L. monocytogenes*, *Pseudomonas aeruginosa*, *Enterococcus* [51,52], thus including also the species tested in the present study. In addition, the effect of carvacrol in terms of cell membrane damage and increased membrane permeability has been recently demonstrated for *L. monocytogenes* Scott A, i.e., the strain used in this study [54]. The use EOs reach in this compound, alone or in combination with other hurdles (physical treatments, other EO constituents, chemical preservatives, inclusion in active packaging) can be a promising strategy to control the growth of the pathogens described above but also the spoilage microflora in food systems [55]. Concerning cinnamon, its strong antimicrobial activity is due to cinnamaldehyde, whose activity against bacteria (such as *Bacillus cereus*, *Campylobacter jejuni*, *Clostridium perfringens*, *E. coli*, *L. monocytogenes* and *Salmonella* enterica), both in model and food systems, has been widely demonstrated [56,57]. Conversely to carvacrol, the antagonistic effect of cinnamaldehyde seems not to be due to cell membrane disintegration but related to the to interactions with membranes inducing damages, such as leakage of some cellular constituents, modification of the proton motive force, and inhibition of membrane bound ATPases [58]. Moreover, some studies reported the efficacy of cinnamaldehyde to reduce the biofilm formation or even to eradicate already established biofilms on different plastic surfaces, in the species *E. coli*, *Staph. aureus*, *Pseudomonas aeruginosa*, *Salmonella* Typhimurium [56]. Eugenol was responsible for the inhibitory effect of clove EO against the target microorganisms. This compound is known to exert insecticidal, antimicrobial, antiviral, anti-inflammatory and antioxidant activity [24]. Concerning antimicrobial effects, different mechanisms of action are reported in literature, including disruption of cell membrane with consequent increased membrane permeability and loss of cellular content, alteration in membrane fatty acid profile, oxidative stress (ROS production), perturbation of the transport of ions and ATP, inhibition of bacterial enzymes [59]. Also, in the case of cumin EO, its efficacy, mainly against *Staph. aureus* was due to the predominance of cuminaldehyde, accounting for one-third of the total peak area. This compound has been reported to induce cell membrane damage, affecting its integrity and thus increasing permeability (also because of its high lipophilic distribution coefficient), to change cell morphology and to bind to DNA, thus interfering with biological functions and inhibiting cell growth [60].

It is noteworthy that the comparison between the MIC of plant EOs from different studies is quite difficult, because of the highly variable chemical composition (due to geographical origin, method of the extraction, part of the plant used) and the use of different testing procedures adopted, i.e., agar diffusion test or broth dilution assay [28,61].

In addition, the effect of chemico-physical parameters on EOs activity has been poorly investigated [62]. Concerning pH, it is generally stated that the susceptibility of bacteria to EOs is higher when pH decreases, even if acid-tolerant species are present [7,63]. Indeed, previous works reported that at pH 5 the antimicrobial activity against *L. monocytogenes* of some EOs, including oregano and marjoram, was higher with respect to neutral values in growth medium and food model media [63,64]. This can be explained by the fact that at low pH, EOs constituents remain undissociated and are more hydrophobic, allowing them to dissolve easily in the bacteria cell membrane lipids [62].

The literature concerning the effect of water activity reduction on EOs activity is scarce. Some studies reported that the addition of NaCl can exert both synergistic and antagonistic activities with EOs and their constituents [62]. For example, 1.2% NaCl allowed an increase in the clove EO antimicrobial effect against *E. coli* [65], while no effects for cinnamaldehyde (the main constituent of cinnamon EO) against Gram-positive (including *Staph. aureus*) or Gram-negative bacteria were observed with the addition of 4% NaCl [66]. Interestingly, a study on essential oils from Lamiaceae EOs reported enhanced antimicrobial activity against *E. coli* in the presence of 3% NaCl when commercial EOs were used, compared to those extracted at laboratory scale. The latter showed lower concentrations of carvacrol, which may explain the reduced efficacy [67]. A synergistic effect between NaCl and oregano essential oil has also been demonstrated by other authors against *Staphylococcus aureus*, using a mathematical model based on response surface methodology (RSM) [68].

## 3. Materials and Methods

### 3.1. Essential Oils

The ten commercial essential oils (EOs) purchased on the market and analyzed in this study are reported in Table 4.

### 3.2. Characterization of EOs Through GC-MS Analysis

The composition of EOs was analyzed using an Agilent 7890A GC System gas-chromatograph and an Agilent 5975C GC/MSD MS detector (Agilent Technologies Italia Spa, Milano, Italy) equipped with a DB-5 60 m × 0.25 mm × 0.25 µm column (Agilent Technologies Italia Spa). The EOs were resuspended in hexane and 1 µL was injected into the following gas chromatographic conditions: injection temperature 250 °C; interface temperature 280 °C; ion source 230 °C; carrier gas (He) flow rate 1.1 mL/min; splitting ratio 1:35. The oven temperature was programmed as follows: 100 °C for 1 min; from 100 to 155 °C with a 5 °C/min rate of increase; 155 °C for 1 min; from 155 to 240 °C with a 30 °C/min increase, then holding for 7 min. The compounds were identified by comparing their spectra with those reported in the NIST 11.0 library (US National Institute of Standards and Technology, Gaithersburg, MD 20899, USA). For each sample, the EO composition was expressed as a relative percentage of each single peak area to the total peak area. Data reported are the means of three repetitions. Only the compounds whose peak area was higher than 0.5% of the total peak area in at least one sample are reported in the tables.

### 3.3. Antioxidant Activity

The DPPH radical scavenging activity of the extracts was evaluated using 96-well microplates [69]. A 290 μL solution of DPPH radical with an initial absorbance of 1.2 was pipetted into the wells, and the absorbance was measured at 517 nm. Then 10 μL of the sample was added, and the reduction in absorbance was measured after 1 h using a plate reader. The antioxidant activity was calculated as the percentage inhibition of the DPPH radical (% inhibition).

The reducing activity was assessed using the FRAP assay (ferric reducing/antioxidant power) [70]. Briefly, 300 μL of the FRAP reagent was dispensed into microplate wells, and the initial absorbance at 592 nm was recorded. Afterward, 10 μL of the sample was added, and the absorbance change was monitored after 4 min. The difference in absorbance between the reaction mixture (after 4 min) and the baseline FRAP reagent was compared to a Trolox standard curve. The results were expressed as millimoles of Trolox equivalents per liter of extract (mM TE/L).

The antioxidant capacity was also determined using the ORAC method, which measures the ability to inhibit peroxyl radicals generated from the decomposition of 2,2-azobis (2-methylpropionamide)-dihydrochloride (AAPH) in the presence of fluorescein [71,72]. Briefly, 150 μL of fluorescein and 25 μL of the sample, Trolox (as the standard), or buffer (blank) were pipetted into the wells and incubated at 37 °C for 30 min. After incubation, 25 μL of AAPH was added, and fluorescence readings were taken every minute for 80 min at excitation and emission wavelengths of 485 nm and 520 nm. The results were expressed as millimoles of Trolox equivalents per liter of extract (mM TE/L).

### 3.4. Bacterial Strains and Growth Conditions

The strains used in this study were *L. monocytogenes* Scott A, *Staph. aureus* DSM 20231^t^, *E. coli* 555 and *Ent. faecalis* EF37, belonging to the collection of the Department of Agricultural and Food Sciences (University of Bologna). The strains were maintained in BHI medium (Oxoid, Basingstoke, UK) with 20% (*w*/*v*) glycerol at −80 °C and, before the experiments, pre-cultivated at 37 °C for 24 h in BHI medium.

### 3.5. Determination of Minimum Inhibitory Concentration (MIC) and Minimum Bactericidal Concentration (MBC)

The *in vitro* antimicrobial activity of these EOs against the target strains was assessed with the broth microdilution method using 96-well microtiter plates (Sarstedt AG & Co., Ltd. KG, Nümbrecht, Germany), following the procedure reported by Barbieri et al. [4]. For the determination of cell growth/no growth, 198 µL of Mueller-Hinton broth (MH; Oxoid, Basingstoke, UK) at pH 7 were inoculated, separately, with each target microorganism at a concentration of about 5 log CFU/mL into 200 µL microtiter wells. EOs were dissolved in ethanol, and 2 µL of these solutions were added to each well to obtain final concentrations ranging between 0.05 and 5 mg/mL. A control well without any EO addition was also performed. To evaluate the impact of chemico-physical parameters on their bioactivity, the same analyses were conducted in MH broth, acidified to pH 6 and 5 by adding a 1 N HCl solution. Moreover, the effect of different salt concentrations was also tested. In particular, 3% or 5% (*w*/*w*) NaCl was added in MH broth, reaching an a_w_ value of 0.982 and 0.970, respectively. Unmodified medium was characterized by an a_w_ of 0.993. Microtiter plates were incubated at 37 °C. The tests were performed in triplicate. The MIC was defined as the lowest concentration of the EO preventing visible growth in the well after 48 h of incubation, while the MBC was defined as the lowest concentration of the EO that caused the death of the inoculated cells, with no growth after 24 h of incubation at 37 °C of a 10-µL spot plated onto BHI agar.

### 3.6. Statistical Analysis

The data of chemical composition obtained through GC-MS were analyzed with OriginPro, Version 2025 (OriginLab Corporation, Northampton, MA, USA).

The statistical difference between the antioxidant activity of the EOs was analyzed using STATGRAPHICS^®^ Centurion XVI (StatPoint Technologies, Inc., The Plains, VA, USA). The variance (one-way ANOVA) procedure at a *p*-value of <0.05 was performed, followed by Fisher’s least significant difference.

## 4. Conclusions

This work studied the composition and bioactive potential of several EOs obtained from Mediterranean officinal plants and spices to evaluate their use in foods as natural preservatives to improve safety and shelf-life. The analyses performed highlighted strong antioxidant activity for cloves due to the predominance of eugenol as the main EO constituent. Regarding antimicrobial activity, cinnamon and oregano exhibited the strongest *in vitro* inhibitory effects against the tested microorganisms, with variations in MIC values observed in relation to pH and water activity. Also in this case, the presence of few compounds in high amounts (cinnamaldehyde and carvacrol, respectively) was responsible for the antagonistic effect and the EO antimicrobial potential was enhanced by the reduction of pH and water activity, showing interesting interactive effects. These observations could lead to the optimization of the use of EOs, also in the perspective of reducing their impact on product organoleptic profile in a frame of hurdle technology. Nowadays, several limitations to EOs use are present, such as interaction with food constituents (lipids, proteins etc.), potential negative impact on product organoleptic characteristics and influences of process and intrinsic variable on their activities.

The findings of this work contribute to the understanding of the potential applications of plant-derived EOs in food preservation. This could offer the food industry a natural, sustainable, and consumer-friendly approach to improving food quality and safety. Further research is needed to better investigate the EOs action mechanism of action and the possible synergic effects with process parameters and product intrinsic characteristics to study their implementation in food productions for an overall sustainability increase in the food sector.

## Figures and Tables

**Table 1:** Chemical composition of the ten commercial essential oils purchased on the market. The data are expressed as relative percentages of each single peak area with respect to the total peak area. Only compounds detected in amounts higher than 0.5% in at least one sample are reported.

Compound (%)	R.T. ^#^ (min)	Cinnamon	Cloves	Cumin	Fennel	Juniper	Laurel	Marjoram	Myrtle	Oregano	Sage
α-phellandrene	5.791	0.65	- *	0.40	11.90	1.53	0.39	2.47	0.22	0.97	0.30
α-pinene	5.948	1.14	-	0.67	13.11	30.89	4.54	0.99	23.33	0.68	4.23
Camphene	6.194	0.57	-	0.02	0.13	0.20	0.27	0.03	0.06	0.08	4.35
β-phellandrene	6.455	2.03	-	0.27	1.54	15.16	9.32	10.61	0.04	0.13	0.46
β-myrcene	6.538	0.05	-	0.27	1.32	12.68	1.26	1.57	0.15	0.98	0.88
β-pinene	6.590	0.36	-	12.78	1.15	2.25	4.05	0.71	0.33	0.22	4.19
4-carene	7.111	0.52	-	0.08	-	0.91	0.41	8.91	-	0.98	-
p-cymene	7.231	1.41	-	12.22	0.83	0.36	0.30	3.60	0.80	7.36	0.98
trans-β-ocimene	7.263	-	-	-	0.53	-	-	-	-	-	0.12
D-limonene	7.325	0.75	-	0.34	9.68	3.63	1.36	2.53	10.61	0.16	1.48
Eucalyptol	7.423	0.27	-	0.19	0.05	-	39.17	0.21	34.31	0.07	10.76
γ-terpinene	7.823	0.09	-	13.16	0.42	1.55	0.83	14.15	0.33	5.09	0.07
β-terpineol	8.017	-	-	0.10	-	0.05	0.31	14.39	-	0.22	0.12
Terpinolene	8.422	-	-	0.05	-	1.36	0.16	3.49	-	-	0.09
Linalool	8.457	1.42	-	0.03	0.09	-	5.89	2.20	4.04	0.95	0.44
L-fenchone	8.521	-	-	-	5.41	-	-	-	-	-	-
Thujone	8.855	-	-	-	-	-	-	-	-	-	35.48
trans-p-Menth-2-en-1-ol	9.127	-	-	-	-	0.04	0.05	0.67	-	-	-
Camphor	9.763	0.69	-	-	0.09	-	-	-	-	0.03	11.72
α-terpineol	10.108	0.63	-	0.13	0.03	0.10	0.89	4.21	3.70	0.10	0.13
endo-borneol	10.184	0.13	-	-	-	-	0.19	0.04	-	0.26	2.70
Terpinen-4-ol	10.382	0.35	-	0.26	0.04	1.22	1.68	24.33	0.18	0.58	0.36
Estragole	10.742	-	-	-	1.68	-	-	0.06	0.19	-	-
Myrtenol	10.806	-	-	-	-	-	-	-	0.51	-	-
Fenchyl acetate	11.314	-	-	-	0.72	-	-	-	-	-	-
Cuminaldehyde	11.870	-	-	32.61	-	-	-	-	-	-	-
trans-cinnamaldehyde	12.621	62.02	-	-	-	-	-	-	-	-	-
Thymol	12.766	-	-	-	-	-	-	-	-	2.66	
Bornyl acetate	12.924	-	-	-	0.05	0.25	-	-	-	-	1.47
Anethole	12.925	-	-	-	50.86	-	-	-	-	-	-
γ-terpinen-7-al	12.994	-	-	14.91	-	-	-	-	-	-	-
α-terpinen-7-al	13.082	-	-	9.79	-	-	-	-	-	-	-
Carvacrol	13.147	-	-	-	-	-	-	-	-	75.93	-
ψ-Limonene	13.589	-	-	-	-	-	0.58	-	-	-	-
Myrtenyl acetate	13.785	-	-	-	-	-	-	-	14.60	-	-
Terpineol acetate	14.215	-	-	-	-	0.07	15.20	-	0.36	-	-
α-cubebene	14.320	-	-	-	-	0.56		-		-	0.03
Eugenol	14.339	4.48	75.20	-	-	-	4.18	-		-	
Geranyl acetate	14.600	-	-	-	-	-		0.04	3.35	-	0.02
Methyleugenol	14.971	-	-	-	-	-	3.27	-	1.02	-	-
β-elemene	14.995	-	-	0.27	-	2.36	0.53	-		-	-
Caryophyllene	15.493	3.01	4.94	0.06	0.06	1.74	1.04	1.51	0.36	1.38	4.32
γ-elemene	15.550	-	-	0.12		2.22	-	-	-	-	-
Acetic acid, cinnamyl ester	15.579	14.09	-	-	-	-	-	-	-	-	-
Humulene	15.910	0.67	0.60			1.78	0.31	0.07	0.19	0.10	8.12
β-copaene	16.211	-	-	0.03	0.18	7.41	-	0.08	-	-	0.09
α-muurolene	16.362	-	-	-	-	1.45	-	-	-	-	-
Eugenol acetate	16.507	-	18.45	-	-		-	-	-	-	-
δ-cadinene	16.545	-	0.07	-	-	4.20	0.13	-	-	-	-
α-amorphene	16.547	0.75	-	-	-	1.41		-	-	-	-
Ledol	17.445	-	-	-	-	-	-	-	-	-	3.90
o-menth-8-ene	17.615	-	-	-	-	-	-	-	-	-	1.30
Benzyl benzoate	18.992	1.07	-	-	-	-	-	-	-	-	-
Total compounds		97.14	99.26	98.75	99.87	95.39	96.34	96.87	98.69	98.92	98.14

^#^: retention time. *: not detected.

**Table 2:** MIC and MBC of the ten EOs against food-borne pathogens and toxigenic bacteria in relation to medium pH. The values are expressed in mg/mL.

Essential Oil (mg/mL)	pH	*Listeria monocytogenes* Scott A		*Staphylococcus aureus DSM 20231^t^*		*Escherichia coli* 555		*Enterococcus faecalis* EF37	
		MIC	MBC	MIC	MBC	MIC	MBC	MIC	MBC
Cinnamon	7	0.25	0.50	0.25	0.50	0.25	0.50	0.50	1
	6	0.25	0.50	0.25	0.50	0.25	0.50	0.50	1
	5	0.10	0.25	0.25	0.50	0.25	0.25	0.50	1
Cloves	7	1	1	1	1	1	1	4	5
	6	1	1	1	1	1	1	2	5
	5	0.25	1	0.25	0.25	1	1	1	2
Cumin	7	>5	>5	4	5	>5	>5	>5	>5
	6	4	>5	3	3	>5	>5	>5	>5
	5	2	>5	1	1	>5	>5	>5	>5
Fennel	7	>5	>5	>5	>5	>5	>5	>5	>5
	6	>5	>5	>5	>5	>5	>5	>5	>5
	5	>5	>5	>5	>5	>5	>5	>5	>5
Juniper	7	>5	>5	>5	>5	>5	>5	>5	>5
	6	>5	>5	>5	>5	>5	>5	>5	>5
	5	>5	>5	>5	>5	>5	>5	>5	>5
Laurel	7	>5	>5	>5	>5	>5	>5	>5	>5
	6	>5	>5	>5	>5	>5	>5	>5	>5
	5	4	>5	2	2	>5	>5	>5	>5
Marjoram	7	5	>5	>5	>5	3	4	>5	>5
	6	4	4	>5	>5	3	3	>5	>5
	5	3	3	1	2	3	3	>5	>5
Myrtle	7	>5	>5	>5	>5	>5	>5	>5	>5
	6	>5	>5	>5	>5	>5	>5	>5	>5
	5	>5	>5	>5	>5	>5	>5	>5	>5
Oregano	7	0.25	0.30	0.25	0.30	0.40	0.40	0.40	0.50
	6	0.20	0.25	0.25	0.20	0.30	0.40	0.40	0.40
	5	0.20	0.20	0.15	0.15	0.25	0.25	0.30	0.40
Sage	7	>5	>5	0.75	0.75	>5	>5	>5	>5
	6	>5	>5	1	1	>5	>5	>5	>5
	5	>5	>5	1	1	>5	>5	>5	>5

**Table 3:** MIC and MBC of the ten EOs against food-borne pathogens and toxigenic bacteria in relation to medium a_w_ due to the addition of different amounts of NaCl: 0% corresponding to a_w_ 0.993, 3% corresponding to a_w_ 0.982, 5% corresponding to a_w_ 0.970. The MIC and MBC values are expressed in mg/mL.

Essential Oil (mg/mL)	NaCl	*Listeria monocytogenes* Scott A		*Staphylococcus aureus* DSM 20231^t^		*Escherichia coli* 555		*Enterococcus faecalis* EF37	
	(%)	MIC	MBC	MIC	MBC	MIC	MBC	MIC	MBC
Cinnamon	0	0.25	0.50	0.25	0.50	0.25	0.50	0.50	1
	3	0.25	0.25	0.25	0.25	0.20	0.20	0.30	0.75
	5	0.20	0.20	0.20	0.25	0.10	0.10	0.25	0.50
Cloves	0	1	1	1	1	1	1	4	5
	3	0.75	1	0.50	0.50	0.50	0.50	5	5
	5	0.30	0.75	0.30	0.50	0.20	0.20	5	5
Cumin	0	>5	>5	4	5	>5	>5	>5	>5
	3	>5	>5	5	5	0.20	0.20	>5	>5
	5	1	>5	5	5	0.20	0.20	>5	>5
Fennel	0	>5	>5	>5	>5	>5	>5	>5	>5
	3	>5	>5	>5	>5	>5	>5	>5	>5
	5	>5	>5	>5	>5	>5	>5	>5	>5
Juniper	0	>5	>5	>5	>5	>5	>5	>5	>5
	3	>5	>5	>5	>5	>5	>5	>5	>5
	5	5	>5	>5	>5	>5	>5	>5	>5
Laurel	0	>5	>5	>5	>5	>5	>5	>5	>5
	3	>5	>5	>5	>5	>5	>5	>5	>5
	5	>5	>5	>5	>5	0.25	0.25	>5	>5
Marjoram	0	5	>5	>5	>5	3	4	>5	>5
	3	5	>5	>5	>5	1	1	>5	>5
	5	5	>5	>5	>5	0.50	0.50	>5	>5
Myrtle	0	>5	>5	>5	>5	>5	>5	>5	>5
	3	>5	>5	>5	>5	>5	>5	>5	>5
	5	>5	>5	>5	>5	>5	>5	>5	>5
Oregano	0	0.25	0.30	0.25	0.30	0.40	0.40	0.40	0.50
	3	0.25	0.25	0.25	0.25	0.10	0.10	0.40	0.40
	5	0.15	0.20	0.25	0.25	0.10	0.10	0.40	0.40
Sage	0	>5	>5	0.75	0.75	>5	>5	>5	>5
	3	>5	>5	0.75	0.75	>5	>5	>5	>5
	5	5	>5	0.40	0.40	0.20	0.20	>5	>5

**Table 4:** Commercial EOs are purchased in the market and characterized for their composition and bioactive properties.

EO	Species	Plant Source
Cinnamon	*Cinnamomum verum* formerly *C. zeylanicum*	Bark
Cloves	*Eugenia caryophillata* Thumb.	Leaves
Cumin	*Cuminum cyminum*	Fruits
Fennel	*Foeniculum vulgare* Mill.	Seeds
Juniper	*Juniperus communis* L.	Berries
Laurel	*Laurus nobilis* L.	Leaves
Marjoram	*Origanum majorana*	Flower parts
Myrtle	*Myrtus communis* L.	Flower parts
Oregano	*Origanum vulgare* L.	Flower parts
Sage	*Salvia officinalis* L.	Flower parts

## Data Availability

The data presented in this study are available on request from the corresponding author.
